# Preparation of Ciprofloxacin-Based Carbon Dots with High Antibacterial Activity

**DOI:** 10.3390/ijms24076814

**Published:** 2023-04-06

**Authors:** Huimin Miao, Panyong Wang, Yingge Cong, Wenfei Dong, Li Li

**Affiliations:** 1 School of Biomedical Engineering (Suzhou), Division of Life Sciences and Medicine, University of Science and Technology of China, Hefei 230026, China; 2 CAS Key Laboratory of Biomedical Diagnostics, Suzhou Institute of Biomedical Engineering and Technology, Chinese Academy of Sciences (CAS), Suzhou 215163, China

**Keywords:** nanomaterials, fluorescent, carbon dots, antibacterial, ciprofloxacin

## Abstract

Nowadays, bacterial infections are attracting great attention for the research and development of new antimicrobial agents. As one of the quinolones, ciprofloxacin (CI) has a broad-spectrum, strong antibacterial effect. However, the clinical use of ciprofloxacin is limited by drug resistance. Ciprofloxacin carbon dots (CCDs) with enhanced antibacterial activity and copper-doped ciprofloxacin carbon dots (Cu-CCDs) were synthesized by a simple hydrothermal method. The results of structural analysis and antibacterial experiments show that CCDs and Cu-CCDs have effective antibacterial properties by retaining the active groups of ciprofloxacin (-COOH, C-N, and C-F), and Cu-CCDs doped with copper have a better antibacterial effect. In addition, experiments have shown that Cu-CCDs show excellent antibacterial activity against *E. coli* and *S. aureus* and have good biocompatibility, which indicates that they have great prospects in clinical applications. Therefore, novel modified copper CCDs with broad-spectrum antibacterial activity, which can be used as antibacterial nanomaterials for potential applications in the field of antibacterial drugs, were synthesized in this study.

## 1. Introduction

Bacterial infections have become a major threat to humanity, bringing multiple challenges, such as the emergence of various diseases and the decomposition of food and drugs [1]. Chemical agents and antimicrobials are the mainstays of clinical treatment for bacterial diseases [2]. However, many chemical agents are biotoxic and disrupt normal tissue cells [3]. Many antibiotics have poor solubility, low bioavailability, and require frequent use in large doses. The widespread and imprudent use of antibiotics has led to the emergence of resistant bacteria. This leads to increased morbidity and mortality from bacterial infections [4]. Therefore, in response to the emergence of new and complex resistant microorganisms, there is an urgent need to discover and develop alternative antimicrobial strategies and antimicrobial agents with superior performance and reduced toxicity to prevent and treat infections [5].

As an alternative to antibiotics, nanomaterials have attracted much attention because of their superior performance and adaptability to the environment [1]. Nanoparticles have a number of unique features due to their extremely small size, which can provide them with antimicrobial activity [6], act as bacterial and viral killers, and treat and prevent infectious diseases [7]. Among them, carbon dots (CDs) are a new type of carbon-based nanomaterial. Due to their unique optical properties, excellent biocompatibility, low toxicity, and ease of modification, they have a wide range of applications in the biomedical field, such as bioimaging, photothermal therapy, drug delivery, biosensors, cancer treatment, and antibacterial treatment [8,9]. CDs can penetrate bacteria and inhibit the formation of bacterial biofilms, resulting in cytoplasmic leakage. They can also bind to bacterial DNA and RNA and change their structure [10,11]. Finally, bacterial growth is affected by many factors to achieve the antibacterial effect. The synthesis methods include top-down and bottom-up routes. In the bottom-up approach, small organic molecules or polymers that act as precursors are converted into CDs with a core–shell structure by dehydration, carbonization, and assembly [12]. Shells can be easily passivated or functionalized by organic groups, and surface functional groups can also possess antibacterial properties. The mechanism of antibiotic action involves the destruction of protein synthesis, the interference of nucleic acid transcription and replication, and the inhibition of metabolic pathways. The antibacterial mechanism involved in CD administration is a complex and disparate process from conventional antibiotics [13]. CDs inhibit bacterial growth and destroy cells through complex mechanisms (photothermal sterilization [14], photodynamic sterilization [15,16], and DNA degradation [17]) and are therefore unlikely to develop resistance.

In recent years, researchers have used various bioactive drugs [18], including natural medicines [19], extraction drugs [20], and clinical drugs [21], as carbon sources and have prepared different drug-based CDs by different methods (electrochemical [22], hydrothermal [23], microwave [24], and thermal decomposition [13]). Levofloxacin [25], ibuprofen [26], ampicillin [27], metronidazole (MET) [28], kanamycin sulfate [29], aspirin [30], artemisinin, and gentamicin sulfate have been used as carbon sources to synthesize corresponding CDs. Studies have found that these CDs, synthesized as antimicrobial agents, can improve the efficiency of treating bacterial infectious diseases. Ciprofloxacin (CI) is a synthetic, third-generation quinolone antibacterial drug with broad-spectrum antibacterial activity and good bactericidal activity that is reportedly 2–4 times stronger than norfloxacin and enoxacin. Ciprofloxacin also has excellent antimicrobial activity and pharmacokinetic properties with few side effects. The wide spread of resistant pathogens makes ciprofloxacin increasingly ineffective, making the development of novel antimicrobials imperative [31].

Selecting the right dopant can enhance the biocompatibility, cell permeability, optical properties, and catalytic performance of CDs to ensure their safe and effective application. The introduction of metals into drugs has been shown to have a profound effect on their biological activity, and many drugs have better pharmacological and toxicological properties when administered in the form of metal complexes. There have been related studies using ZnO [32,33], TiO_2_, and other materials with CD synthetic composites to improve bactericidal performance. There have also been studies on the direct synthesis of CDs doped with ruthenium [34], silver [35], gadolinium [36], and copper [37,38,39,40] to improve their performance and enhance their antioxidant and antibacterial activities. Therefore, metal-embedded ciprofloxacin could provide a greater antibacterial effect.

**Scheme 1:**
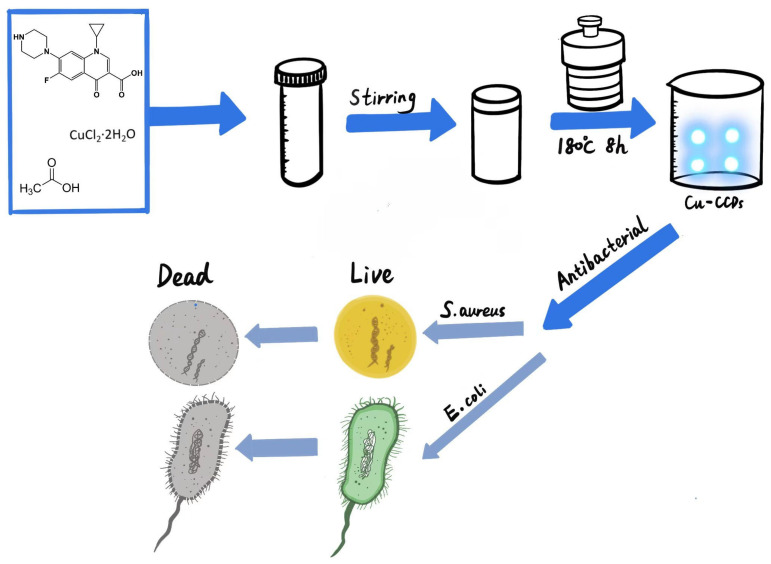
Schematic illustration of the preparation and antibacterial application of Cu-CCDs.

In this work, ciprofloxacin-based carbon dots (CCDs) with low drug resistance and good solubility and copper-doped ciprofloxacin-based carbon dots (Cu-CCDs) were synthesized by a simple hydrothermal method, and their morphology, optical properties, and antibacterial properties were compared (Scheme 1). In the antibacterial test, Cu-CCDs showed better antibacterial performance against Gram-positive and Gram-negative bacteria compared to CCDs. The minimum inhibitory concentrations of Cu-CCDs against *Escherichia coli* and *Staphylococcus aureus* were 2.5 and 5 μg/mL, respectively. Positively charged CCDs attached to negatively charged bacteria through electrostatic interactions, damaging the bacterial membrane structure and causing the leakage of intracellular material that ultimately killed the bacteria. This confirms the prospects of CCDs in antimicrobial treatments. This work produced a low-toxicity antibacterial agent, which can treat bacterial infections while avoiding drug resistance. The most significant findings of this work highlight the drug-based synthetic design and synergistic antibacterial mechanism, including the process of Cu-CCD bacterial killing without external additional stimulation.

## 2. Results and Discussion

### 2.1. Synthesis and Characterization of CDs

**Figure 1:**
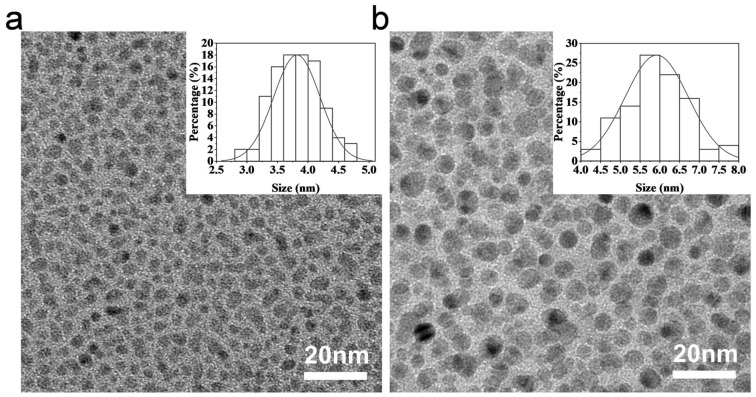
TEM images of the (**a**) CCDs and (**b**) Cu-CCDs.

The CCDs and Cu-CCDs were synthesized by a one-step hydrothermal method, as shown in Scheme 1. With the increase in reaction temperature, the raw material ciprofloxacin molecule is converted into CDs through dehydration, carbonization, and assembly [3]. To analyze the morphology of the synthesized CDs, we characterized them by TEM. As shown in Figure 1, the prepared CDs were spherical in shape and uniformly distributed. These spherical particles range in size from 3 to 8 nm. The average sizes of the produced CCDs and Cu-CCDs were 3.8 nm (Figure 1a) and 5.9 nm (Figure 1b), respectively, counted and calculated from TEM images. The size of Cu-CCDs was larger than that of CCDs due to the coupling of copper to the surface of the ciprofloxacin carbon dot, increasing the Cu-CCD diameter.

**Figure 2:**
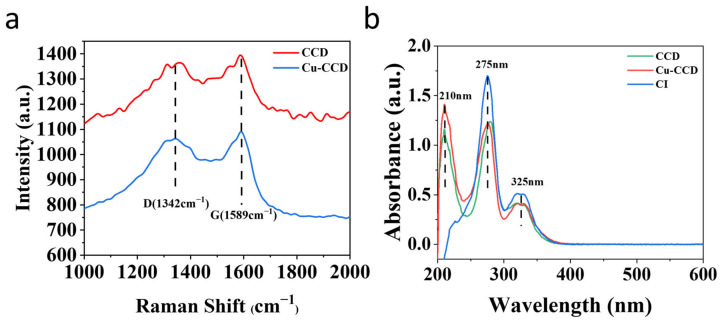
(**a**) Raman spectra of CCDs and Cu-CCDs; (**b**) The UV–vis spectra of the CI, CCDs, and Cu-CCDs.

The Raman spectra of CCDs and Cu-CCDs (Figure 2a) show two peaks at 1342 cm^−1^ (D-band) and 1589 cm^−1^ (G-band), where the D-band indicates defects in the lattice and the G-band indicates in-plane stretching vibrations. The ID/IG of synthetic carbon points is 0.97, less than 1, which proves that they are graphene structures [41]. However, the ID/IG value of ciprofloxacin itself is higher than 1 [42], the ID/IG value decreases significantly, and the transition from disorder to order is complete in the synthesis of carbon dots. As shown in Figure 2b, the UV–vis absorption spectra of aqueous solutions of CCDs and Cu-CCDs show strong absorption peaks at 210 nm and 275 nm, which originate from π-π* leaps in the aromatic sp^2^ structural domain (C=C, C-C) [43]. The weak peak at 325 nm is derived from the n-π* leap of the C=O and C-N bonds [38]. However, the raw material CI only shows one strong peak at 275 nm, attributed to the π-π* leap (C=C, C-C). This also indicated that the synthesized CDs retained the ring structure of ciprofloxacin [44]. These results are consistent with other reports [45,46].

**Figure 3:**
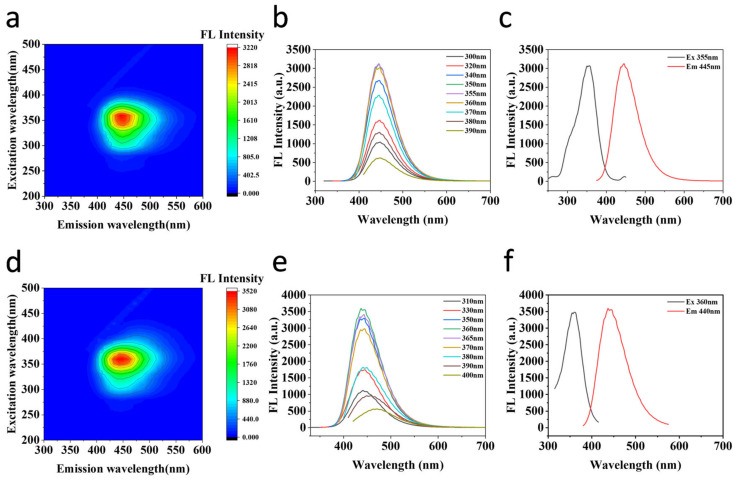
(**a**) Excitation–emission—matrix spectra of the CCDs; (**b**) excitation and emission spectra of CCDs; (**c**) emission spectra of CCDs, increasing the excitation wavelength from 300 nm to 390 nm. (**d**) Excitation–emission—matrix spectra of Cu-CCDs; (**e**) excitation and emission spectra of Cu-CCDs; (**f**) emission spectra of Cu-CCDs, increasing the excitation wavelength from 310 nm to 400 nm.

Further studies showed that CCDs and Cu-CCDs have good optical properties and high solubility in water. The fluorescence spectra with different excitation wavelengths are shown in Figure 3. The spectra exhibit characteristic excitation-dependent fluorescence features at 310 nm to 380 nm. The CCDs exhibited the greatest emission at 445 nm under 355 nm excitation. The Cu-CCDs exhibited the greatest emission at 440 nm under 360 nm excitation, where Cu-CCDs emitted a stronger fluorescence than CCDs [47]. This may be because the conjugate structure in ciprofloxacin can interact with Cu^2+^. The copper’s energy level is lower than that of the host CDs’ excited state. Therefore, the excited-state electrons of the CDs transfer to the copper energy level to form a new FL emission [38].

**Figure 4:**
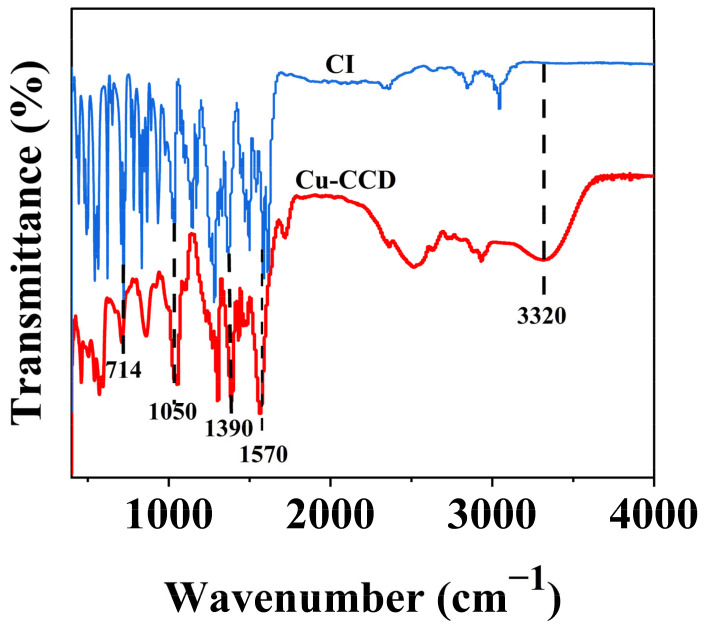
FTIR spectra of CI and Cu-CCDs.

To further explore the properties of Cu-CCDs, the surface groups of the Cu-CCDs were investigated by FTIR and XPS. Figure 4 shows the FTIR spectrum of Cu-CCDs, and the analysis of the transmission peak intensities shows that the surface of the carbon dots is rich in hydroxyl and carbonyl groups. The characteristic absorption bands located around 714 cm^−1^ and 1390 cm^−1^ are due to the stretching vibrations of the C-N group. The peaks at 1050 cm^−1^ and 1570 cm^−1^ are due to the stretching vibrations of C-O and C=O, respectively. In addition, the absorption peak at 3320 cm^−1^ is due to the stretching vibrations of the -OH bond, indicating the excellent water solubility of the sample. Moreover, the above results also indicate the existence of a carboxyl group (-COOH), attributed to the symmetric and asymmetric stretching vibrations of the O=C-O group [48]. These oxygen-containing groups provide the excellent dispersion ability of the Cu-CCDs in solution. The results of Fourier transform infrared spectroscopy confirmed the presence of hydroxyl and carbonyl groups on the surface of the CDs, indicating their successful synthesis.

**Figure 5:**
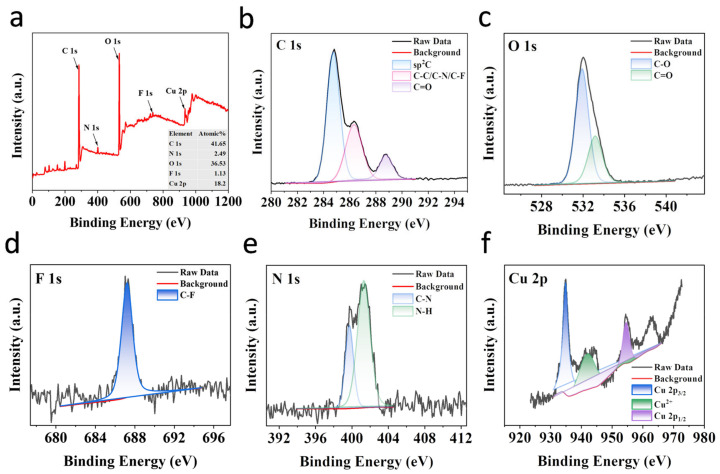
XPS full scan spectra of Cu-CCDs (**a**) and high-resolution C 1s (**b**), O 1s (**c**), F 1s (**d**) N 1s (**e**) and Cu 2p (**f**) XPS spectra of Cu-CCDs.

Elemental analysis showed that the synthesized carbon-dotted Cu-CCDs mainly consisted of C, N, O, F, and Cu. As shown in Figure 5, the C 1s XPS spectra show different peaks at 284.8, 286.3, and 288.7 eV, belonging to sp^2^C, C-C/C-N/C-F, and C=O bonds, respectively [49]. The O 1s XPS spectra show two peaks at 531.9 and 533.2 eV, which are C-O and C=O, respectively [50]. These results agree with the FTIR observations, confirming the formation of carboxyl and carbonyl groups on the nanoclusters. The F 1s spectra show a typical peak at 687.2 eV, which is the C-F bond in the CD structure. The N 1s XPS spectra show two peaks at 399.7 and 401.3 eV for the C-N and N-H bonds, respectively [51]. As shown in Figure 5f, the peak at around 954.6 eV corresponds to Cu 2p_1/2_, and the peak at 934.8 eV belongs to Cu 2p_3/2_. Observable satellite peaks (939–944 eV) were assigned to Cu (II) [52]. These XPS spectra results indicate that the Cu-CCDs are composed of carbonyl, carboxylic, F-containing, and N-containing groups as well as Cu. On the basis of the above analysis, we conclude that Cu-CCDs inherit the active groups of ciprofloxacin and acetic acid, including -COOH, C-N, and C-F, which provide assurance for good solubility and a positively charged surface.

### 2.2. Cytotoxicity Analysis

**Figure 6:**
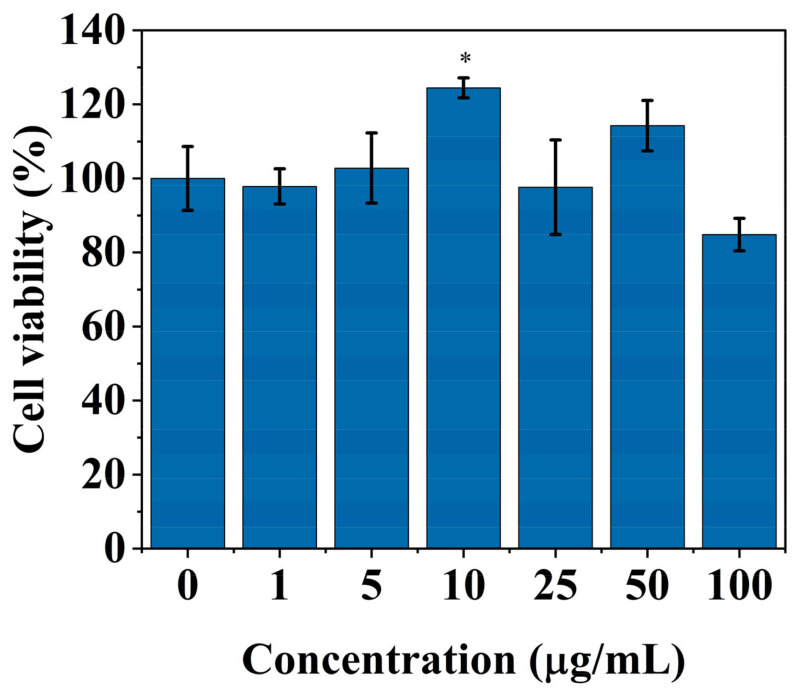
Relative viability of 3T3 cells treated with different concentrations of Cu-CCDs (0, 1, 5, 10, 25, 50, 100 μg/mL). Significance analysis was carried out by one-way ANOVA. * *p* < 0.05, *n* = 3, compared to the control group (0 μg/mL). The statistically significant differences were found only between the 0 μg/mL sample and 10 μg/mL sample.

Before using the synthesized Cu-CCDs for further biological applications, the biocompatibility of 3T3 cells was investigated by WST-1. As shown in Figure 6, the cell survival rate was above 80% after co-incubation of Cu-CCD solutions at different concentrations (0–100 μg/mL) for 24 h. When the concentration is below 50 μg/mL, the cell survival rate is almost 100%. At such high concentrations, cell growth was not inhibited, demonstrating that the addition of Cu-CCDs had no significant impact on cell growth. Therefore, the Cu-CCDs had negligible toxicity to the 3T3 cells in vitro. Thus, it was confirmed that the synthesized Cu-CCDs have low toxicity and good biocompatibility.

### 2.3. Antibacterial Performance

#### 2.3.1. Spread Plate Method

**Figure 7:**
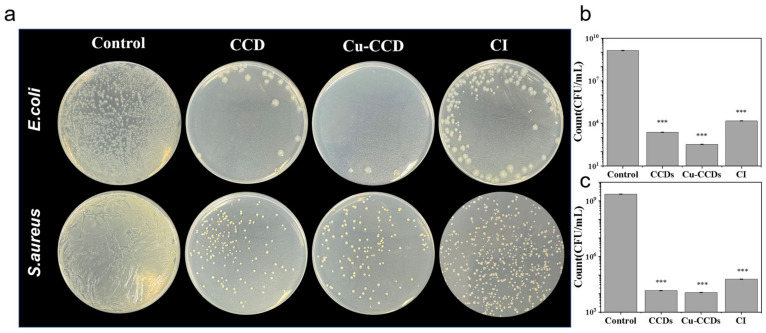
(**a**) Typical photos of *E. coli* and *S. aureus* after 24 h of treatment with different CDs and CI on LB nutrient agar medium; (**b**,**c**) statistical histograms of bacterial colonies counted from the LB plates. Significance analysis was carried out by one-way ANOVA. *** *p* < 0.001, *n* = 3, compared to the control group.

We used the spread plate method to assess the antibacterial activity of CCDs and Cu-CCDs. The susceptibility of bacteria to high drug concentrations inhibited their growth on LB nutrient agar, and the antimicrobial performance of CDs was evaluated based on the number of colonies formed on the LB plates. As shown in Figure 7a, the LB plates without CDs were full of colonies, while fewer colonies formed on the LB plates with CCDs and Cu-CCDs. Specific statistical data can be seen in Figure 7b,c. Quantification of the *E. coli* in the LB plates showed 1.35 × 10^9^ CFU/mL for the control group. After treatment with CCDs and Cu-CCDs, the bacterial count on the plate was 2.3 × 10^3^ CFU/mL and 3.3 × 10^2^ CFU/mL, respectively. The inhibition effect of Cu-CCDs was more significant (with a bacterial killing efficiency of 99.9%). Similarly, the quantification of the *S. aureus* in the LB plates showed 2.32 × 10^9^ CFU/mL for the control group. Differently, *S. aureus* treated with Cu-CCDs resulted in a bacterial burden of 1.1 × 10^4^ CFU/mL, a significant reduction in the bacterial number (with a bacterial killing efficiency of 99.9%) as compared to the control group. In summary, the antibacterial effects of CCDs and Cu-CCDs are better than those of ciprofloxacin, and the bactericidal effects of Cu-CCDs are more significant. This may be because of the antibacterial effect of copper itself. After being mixed into CDs, copper can cooperate with the CDs to resist bacteria. It is noteworthy that CDs had a better inhibition effect on *E. coli* than *S. aureus*.

#### 2.3.2. MIC of Cu-CCDs

**Figure 8:**
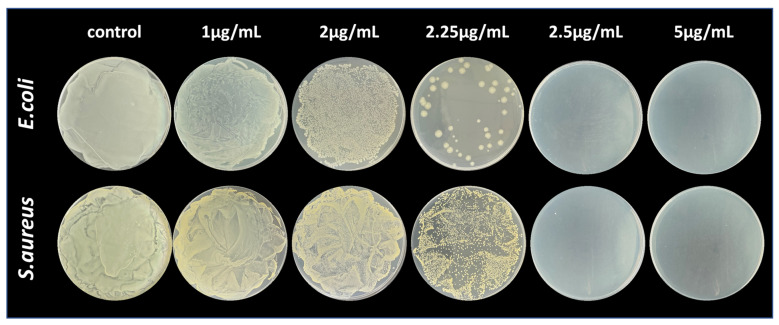
Typical photographs of *S. aureus* and *E. coli* after treatment with different concentrations of Cu-CCDs (0, 1, 2, 2.25, 2.5, and 5 μg/mL) for 24 h.

To determine the minimum inhibitory concentration (MIC) of Cu-CCDs, *E. coli* and *S. aureus* were co-cultured with different concentrations of Cu-CCD solutions (0, 1, 2, 2.25, 2.5, and 5 μg/mL) in LB agar medium. The results are shown in Figure 8. When the concentration of CDs increased, the number of colonies that formed on the plates decreased, and when the concentration of Cu-CCDs reached 5 μg/mL, no colonies were generated for *E. coli* or *S. aureus*. This showed that Cu-CCDs at 5 μg/mL could inhibit all pathogenic microorganisms with high efficiency. These results agree with the spread plate method data, confirming the effective antibacterial activity of Cu-CCDs.

#### 2.3.3. Bacterial Morphology Studies

**Figure 9:**
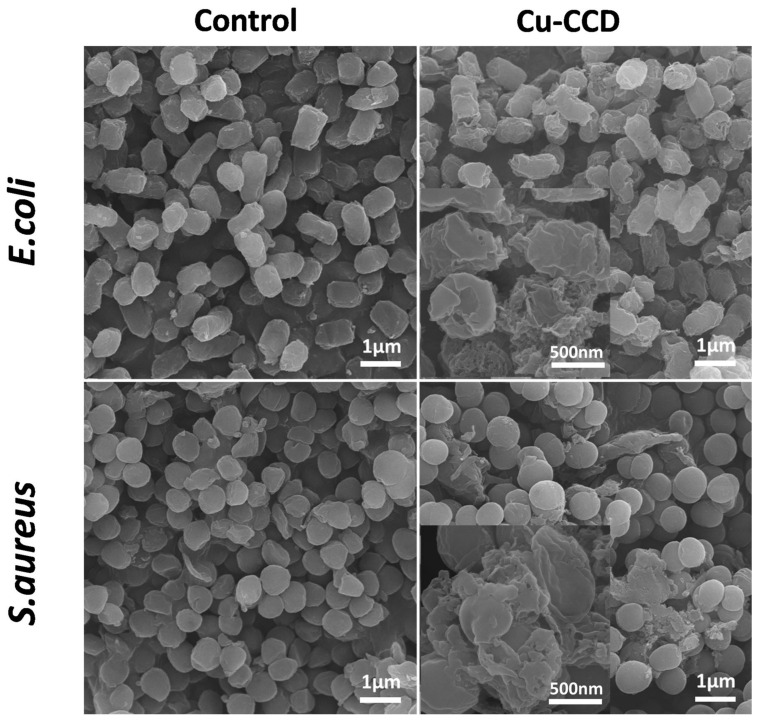
SEM images of *E. coli* and *S. aureus* treated with Cu-CCDs.

The SEM morphology of *E. coli* and *S. aureus* treated with Cu-CCDs is given in Figure 9. As shown in the figure, the cell surfaces of untreated *E. coli* and *S. aureus* were intact and smooth; after treatment with Cu-CCDs, the cell surfaces of *E. coli* and *S. aureus* showed wrinkling or perforation, wall and membrane rupture, intracellular material leakage, and obvious cell damage [23,53]. The results indicated that Cu-CCDs caused damage to the surfaces of *E. coli* and *S. aureus*, similar to the action of cationic antimicrobial agents [54]. The above results also demonstrate the excellent potential of Cu-CCDs in antibacterial applications. 

#### 2.3.4. Zeta Potential Measurements

**Figure 10:**
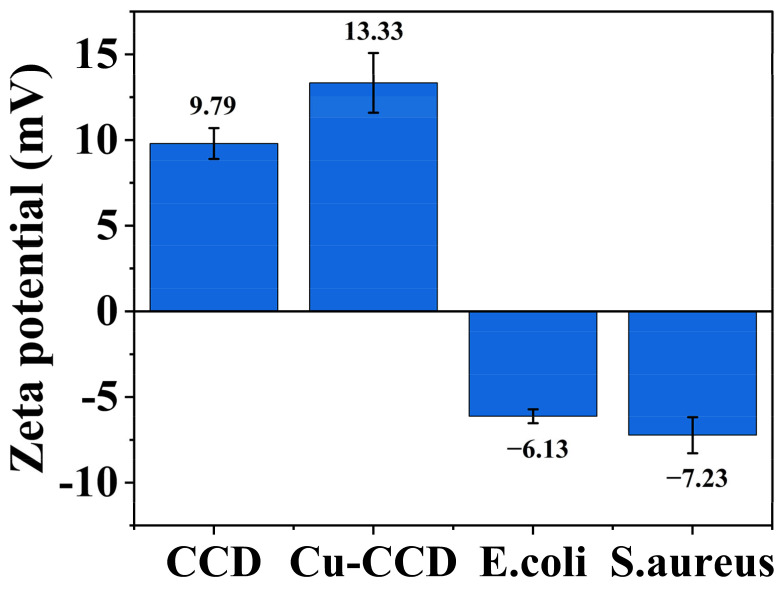
Zeta potential of bacterial and synthetic CDs.

As shown in Figure 10, the zeta potentials of both CCDs and Cu-CCDs are positive, while those of both *E. coli* and *S. aureus* are negative. The zeta potential of Cu-CCDs is higher than that of CCDs, this may be due to Cu^2+^ doping increasing the number of surface positive charges. It can be inferred that CDs are bound to the bacterial surface through electrostatic interactions, which in turn destroy the bacterial structure, thus killing the bacteria [55]. Traditional antibiotics were easily discharged from the bacteria, leading to resistance, while CCDs and Cu-CCDs attached to the bacteria via electrostatic interactions; therefore, they were capable of physically damaging the bacterial cell membrane and avoiding resistance. 

## 3. Materials and Methods

### 3.1. Materials

Ciprofloxacin (CI) was purchased from Shanghai Aladdin Biochemical Technology Co., Ltd., (Shanghai, China). Acetic acid was purchased from Shanghai Titan Scientific Co., Ltd., (Shanghai, China). Copper chloride dihydrate was purchased from Thain Chemical Technology Co., Ltd., (Shanghai, China). The WST-1 cell proliferation and cytotoxicity assay kit was purchased from Beyotime Biotechnology Co., Ltd., (Shanghai, China). RPMI Medium 1640 basic (1×) was purchased from Thermofisher Biochemical Products (Beijing, China) Co., Ltd. Luria Bertani broth and Luria Berdani agar were obtained from Qingdao Hope Bio-Technology Co., Ltd., (Qingdao, China). *Escherichia coli* (ATCC 25922) and *Staphylococcus aureus* (ATCC 6538) were purchased from Beijing Preservation Biotechnology Co., Ltd., (Beijing, China). NIH-3T3 cells (mouse embryonic fibroblasts) were purchased from Shanghai FuHeng Biology Co., Ltd., (Shanghai, China).

### 3.2. Synthesis of CCDs and Cu-CCDs

CDs were synthesized through a one-step hydrothermal method using ciprofloxacin as a precursor. In total, 0.01 g of ciprofloxacin was dissolved in 5 mL of acetic acid, and then 25 mL of deionized water was added to the obtained solution. A clear solution was obtained by ultrasonic oscillation for 10 min. The solution was heated at 180 °C for 8 h in a 100 mL stainless-steel autoclave lined with PTFE. The obtained solution was the crude product CCDs. Cu-CCDs were synthesized in a similar way. In total, 0.01 g of ciprofloxacin and 0.05 g of copper chloride dihydrate were dissolved in 5 mL of acetic acid and 25 mL of ultrapure water. Then, a clear solution was obtained by ultrasonic oscillation for 10 min. This solution was then heated at 180 °C for 8 h [56]. Subsequently, the reacted solution was cooled to room temperature, and the product was dialyzed for 2 h through a 1000 Da dialysis membrane with deionized water to remove unreacted ciprofloxacin. Finally, the CD powder was obtained by rotary evaporation. The powder was dissolved in deionized water to obtain a 5 mg/mL solution of CD to be used.

### 3.3. Characterization Methods

The morphologies of CCDs and Cu-CCDs were recorded on a transmission electron microscope (TEM, JEOL Ltd., Tokyo, Japan). Fluorescence spectra and UV–visible absorption spectra were collected with a fluorescence spectrophotometer (F97Pro) and a U-3900H spectrophotometer (Hitachi, Tokyo, Japan). Raman spectra were acquired on a confocal Raman spectrometer (HOOKE P300, HOOKE INSTRUMENTS Ltd, Jilin, China). Zeta potential measurements were investigated using a Nano ZS/ ZEN3690 (Malvern, UK). Energy dispersive X-ray spectra were recorded on an AXIS ULTRA DLD spectrometer (Escalab 250xi, TMO, Waltham, MA, USA). The Fourier transform infrared (FTIR) spectra were acquired on an FT-IR spectrometer (Agilent Cary 660, Santa Clara, CA, USA). The morphological bacterial changes were monitored by scanning electron microscopy (SEM, Hitachi, Tokyo, Japan).

### 3.4. Cytotoxicity Analysis

We performed cytotoxicity experiments with NIH-3T3 cells (mouse embryonic fibroblasts) at different concentrations of Cu-CCDs. Cytotoxicity tests were performed with the WST-1 cell proliferation and cytotoxicity assay kit. NIH-3T3 cells were inoculated in 50 mL cell culture bottles (37 °C, 5% CO_2_). After 24 h, approximately 5 × 10^6^ cells/bottle were obtained, and the cells were diluted with culture medium. The NIH-3T3 cells were added into 96-well plates at a density of 5000 cells per well (100 μL), cultured in an incubator (37 °C, 5% CO_2_) for 24 h, and then incubated with Cu-CCDs solutions of different concentrations (100, 50, 25, 10, 5, and 1 μg/mL) for 24 h. Afterwards, the culture medium was removed from each well, and new medium containing 10 μL of WST-1 was added to each well for 2 h. Finally, the absorbance at 450 nm was recorded with a Synergy HTX Multi-Mode Reader. The wells with 2.5 μL of PBS and 100 μL of 1640 medium were used as a blank control (*OD_blank_*), and the wells with 2.5 μL of PBS and 100 μL of bacterial solution were used as the positive control (*OD_control_*). The cell *survival rate* was calculated with the following formula:

$$Survival\;rate=\frac{{OD}_{Treatment\;group}-{OD}_{Blank}}{{OD}_{Control\;group}-{OD}_{Blank}}\times 100\%$$

### 3.5. Antibacterial Performance

#### 3.5.1. Bacterial Cell Culture

Luria–Bertani (LB) broth and Luria–Bertani agar were used as the medium. The bacteria (*E. coli* and *S. aureus*) were inoculated from an inclined medium and cultured in LB broth at 37 °C and 200 rpm for 24 h. Finally, 1 × 10^9^ CFU/mL of bacterial suspension was obtained for use. The bacterial turbidity meter WGZ-XT was used to determine the initial concentration of bacteria.

#### 3.5.2. Spread Plate Method 

The antibacterial activity of CCDs, Cu-CCDs, and CI against *E. coli* and *S. aureus* was tested by the spread plate method [53]. In total, 50 μL of bacterial suspension (1 × 10^9^ CFU/mL) was added to 5 mL LB broth and incubated with CCDs and Cu-CCDs (10 μg/mL) for 12 h at 37 °C. After this, 10 μL of culture solution was dispersed onto LB nutrient agar and incubated for 24 h at 37 °C. In addition, the group without nanoparticles was set as a positive control. Finally, colony-forming units (CFU) on the plates were counted to compare the antibacterial activity of CCDs and Cu-CCDs. The control group first diluted and then counted the coated plates. Based on the spread plate method, it is indicated that Cu-CCD had better results in inhibiting bacterial growth, and therefore the decision was made to only evaluate MIC, microscopy, and other characterizations for Cu-CCDs and not for CCDs.

#### 3.5.3. MIC of Cu-CCDs

To investigate the bacteriostatic effect of Cu-CCDs against *S. aureus* and *E. coli*, the operational steps were as follows [57]. In total, 50 μL of *S. aureus* and *E. coli* suspension (1 × 10^8^ CFU/mL) and 5 mL of LB broth were incubated with different concentrations (5, 2.5, 2.25, 2, and 1 μg/mL) of Cu-CCDs solution at 37 °C for 12 h. After this, 100 μL of culture solution was dispersed onto LB nutrient agar plates and incubated at 37 °C for 24 h to observe colony formation. We set the Cu-CCDs concentration added to the plate without colony growth as the MIC. The group without nanoparticles was used as the positive control.

#### 3.5.4. Bacterial Morphology Study

The morphology of bacteria before and after treatment with Cu-CCDs was observed by SEM [19]. In total, 50 μL of the above cultured bacterial solution (1 × 10^8^ CFU/mL) was added to 5 mL of LB broth, and 2.5 μL Cu-CCDs solution (5 mg/mL) was added and incubated at 37 °C for 24 h. Subsequently, 100 μL of culture solution was removed and diluted to 1 mL, then the above solution was centrifuged at 1500 rpm for 3 min and the supernatant was discarded to obtain the bacterial precipitate. The condensed bacteria were mixed with 2 mL of 2.5% glutaraldehyde and fixed for 2 h at room temperature. Then, these bacteria were treated stepwise with different concentrations of alcohol solution (30, 50, 70, 80, and 90%). Finally, these dehydrated bacteria were resuspended in 100% alcohol, dropped onto a silicon slice, and then laid flat to dry in the air. The processed samples were used to test the SEM images after gold sputtering treatment. The bacterial solution without Cu-CCDs was set as the control group.

#### 3.5.5. Zeta Potential Measurement

In total, 50 μL of bacteria (*E. coli* and *S. aureus*) were inoculated into 5 mL of LB medium, cultured on a shaking table at 37 °C for 6 h, and then the zeta potential of the bacterial solution was measured. At the same time, the zeta potential of the synthesized CCDs and Cu-CCDs (5 mg/mL) was measured [55].

## 4. Conclusions

In this study, we synthesized CCDs and Cu-CCDs that are more water-soluble than ciprofloxacin and possess excellent fluorescence properties, biocompatibility, and antibacterial activity. Among them, CCDs doped with Cu had better fluorescence and antibacterial performance than CCDs without Cu doping. Moreover, CDs synthesized based on ciprofloxacin can fight drug-resistant bacteria and overcome the limitations of antibiotic treatment. Therefore, Cu-CCDs have the potential to act as new antimicrobial agents. Based on the results of the zeta potential measurement, it can be inferred that the surface of the synthesized CDs is positively charged due to the negatively charged bacterial surface. Therefore, positively charged CCDs and Cu-CCDs attach to the bacterial surface through electrostatic interaction, damaging the bacterial membrane structure and thereby inhibiting bacterial growth. Bacterial morphological studies have shown that Cu-CCDs destroy the cell wall and membrane of bacteria after adsorption onto the bacterial surface, resulting in the outflow of internal materials inside the bacterial cell, causing irreversible damage and killing the bacteria. In summary, we have synthesized a new type of CI-based antibiotic, Cu-CCDs, by using simple hydrothermal reactions. This simple and low-cost preparation method makes it easy to realize large-scale production. This material has great potential in combating bacterial infection and overcoming bacterial drug resistance.

## Data Availability

Data are available on request.
